# Unravelling the Interaction of Piperlongumine with the Nucleotide-Binding Domain of HSP70: A Spectroscopic and In Silico Study

**DOI:** 10.3390/ph14121298

**Published:** 2021-12-13

**Authors:** Ana Paula Ribeiro Povinelli, Gabriel Zazeri, Alan M. Jones, Marinônio Lopes Cornélio

**Affiliations:** 1 Departamento de Física, Instituto de Biociências, Letras e Ciências Exatas (IBILCE), Universidade Estadual Paulista, UNESP, Rua Cristovão Colombo 2265, São José do Rio Preto 15054-000, Brazil; ana.povinelli@unesp.br (A.P.R.P.); gabriel.zazeri@unesp.br (G.Z.); 2 School of Pharmacy, University of Birmingham, Edgbaston, Birmingham B15 2TT, UK

**Keywords:** heat shock protein, HSP70, nucleotide-binding domain, piperlongumine, fluorescence spectroscopy, circular dichroism, molecular docking, molecular dynamics, molecular mechanics Poisson–Boltzmann surface area

## Abstract

Piperlongumine (PPL) is an alkaloid extracted from several pepper species that exhibits anti-inflammatory and anti-carcinogenic properties. Nevertheless, the molecular mode of action of PPL that confers such powerful pharmacological properties remains unknown. From this perspective, spectroscopic methods aided by computational modeling were employed to characterize the interaction between PPL and nucleotide-binding domain of heat shock protein 70 (NBD/HSP70), which is involved in the pathogenesis of several diseases. Steady-state fluorescence spectroscopy along with time-resolved fluorescence revealed the complex formation based on a static quenching mechanism. Van’t Hoff analyses showed that the binding of PPL toward NBD is driven by equivalent contributions of entropic and enthalpic factors. Furthermore, IDF and Scatchard methods applied to fluorescence intensities determined two cooperative binding sites with K_b_ of (6.3 ± 0.2) × 10^4^ M^−1^. Circular dichroism determined the thermal stability of the NBD domain and showed that PPL caused minor changes in the protein secondary structure. Computational simulations elucidated the microenvironment of these interactions, showing that the binding sites are composed mainly of polar amino acids and the predominant interaction of PPL with NBD is Van der Waals in nature.

## 1. Introduction

Living organisms, regardless of their kingdom, are constantly subjected to stressful situations and respond to these stimuli through changes in cellular metabolism, activating their defence mechanisms [1]. The stress response includes heat shock proteins (HSPs), which is one of the primary cellular protection responses [2,3].

HSPs are part of the large family of proteins known as molecular chaperones, so called because they have the ability to interact reversibly with other proteins, helping in formation, folding and trans-membrane transport [4]. HSP70 is the 70 kDa heat shock protein, composed of a conserved N-terminal nucleotide binding domain (NBD) with ATPase activity, a substrate binding domain (SBD) and a C-terminal domain. Among the domains, NBD is a 40 kDa ATP binding domain [5] with a highly flexible chain.

The HSP70 was found to be over expressed in various cancers in response to the stressful environment of tumors, leading to tumor protection and consequently to therapeutic resistance. Recently, the set of functions of heat shock proteins (HSP) has been extended based on studies that have shown that HSP70 is also found in the extracellular environment and exhibits potent cytokine activity, with the ability to activate the nuclear factor-kappaB (NF-κB) and consequently regulate the expression of pro-inflammatory cytokines [6,7,8]. Due to the involvement of HSP70 in the pathogenesis of several diseases, this macromolecule has become a potential molecular target for the development of pharmacotherapies [9]. Much effort has been dedicated to the search for small ligands that are able to inhibit the chaperone function of HSP70, mostly through the interaction with ADP binding site [5,10]. However, to the best of our knowledge the decrease of HSP70 inflammatory activity due to the binding of small ligands has not been investigated.

**Figure 1:**
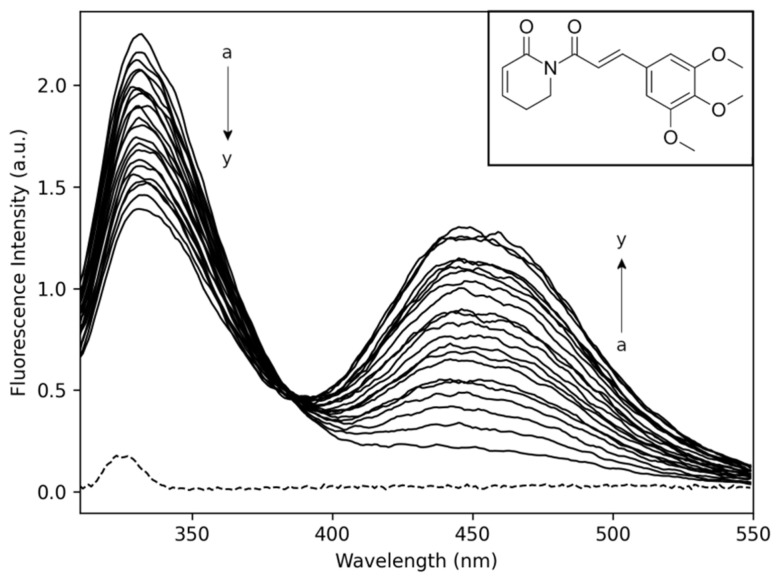
NBD fluorescence emission spectra obtained from titration experiments with increments in the concentration of PPL (pH 7.4, T = 283 K, λ_excitation_ = 295 nm). [*NBD*] = 4.0 μM; PPL titrations with increments of −1.0 μM (a → y = 0 μM → 24 μM). Inset: Chemical structure of PPL.

Piperlongumine (PPL) is a small molecule that has been reported to have a multitude of biological activities such as anti-inflammatory, anti-carcinogenic, anti-atherosclerotic, amongst others [11,12,13,14]. PPL is an alkaloid isolated from long pepper that is widely used in Indian traditional medicine [15,16]. This molecule is characterized by the presence of electrophilic motifs [12] including an α,β-unsaturated amide acting as a Michael acceptor (inset of Figure 1) [17]. As a potential drug scaffold, PPL has no Lipinski or lead-like rule violations [18]. Biological studies have shown that the anti-inflammatory and anti-carcinogenic activities of PPL are a consequence of the inhibition of NF-κB pathway [17,19,20,21]. However, to the best of our knowledge there are no studies to date regarding the molecular interaction of PPL and NBD/HSP70.

In the present work we bring a detailed biophysical characterization of the interaction of PPL with NBD to support further drug discovery efforts. The experimental characterization is based on multi-pronged spectroscopic approaches. Fluorescence and circular dichroism spectroscopy were employed to disclose the number of binding sites, the mode of binding, the binding affinity, the thermodynamic parameters of interaction and the protein conformational changes due to these interactions. To have a complete description of the complex, molecular docking and dynamics parameterized by experimental results were employed to predict the binding sites and to disclose the molecular interactions in the microenvironments.

## 2. Results and Discussion

### 2.1. Fluorescence Spectroscopy

Figure 1 shows the fluorescence emission spectra of Trp90 NBD domain at 330 nm in the absence (a) and the presence of PPL (→y). Furthermore, Figure 1 shows PPL fluorescence emission band at 450 nm while the molecule was added to the solution (→y). The full-width half maximum (FWHM) of ±30 nm for the band of Trp90 and ±45 nm for the band of PPL showed that PPL fluorescence emission intensity did not influence NBD fluorescence emission spectra intensity, which allows for further analyses of the quenching mechanism. The interaction of NBD with PPL was monitored following the intensity of Trp90 emission spectra at 330 nm. Analysis of the spectra revealed the NBD fluorescence was quenched with the addition of PPL in the sample, demonstrating the existence of the quenching effect upon the Trp90 emission.

The quenching mechanism may be classified as dynamic (diffusive encounters) or static (complex formation) processes. It is possible to differentiate them by analysing the dependence of Stern–Volmer constant (*K_SV_*) with temperature (Equation (1)) [22]. In general terms, static quenching reflects a decrease in *K_sv_* with the increase in temperature, while dynamic quenching may provoke an increase in *K_sv_* with the increase in temperature. Another method to determine the quenching mechanism involves comparing the ratio of fluorescence signals (*F*_0_/*F*) with the ratio of the lifetime values (*τ*_0_/*τ*). For dynamic quenching, the relation *F*_0_/*F* = *τ*_0_/*τ* has to be observed for the system, otherwise collisions are not observed and the quenching is static [23]. The analysis of the bimolecular quenching rate constant (*k_q_*) is another method that can also be used to confirm the quenching mechanism. To have the system ruled by collisions (dynamic quenching), the bimolecular constant cannot exceed the limit of 10^10^ M^−1^·s^−1^ [23].

(1) $$\frac{F_{0}}{F}=1+K_{SV}\cdot \left[PPL\right]=1+k_{q}\cdot \tau_{0}\cdot \left[PPL\right]$$

**Figure 2:**
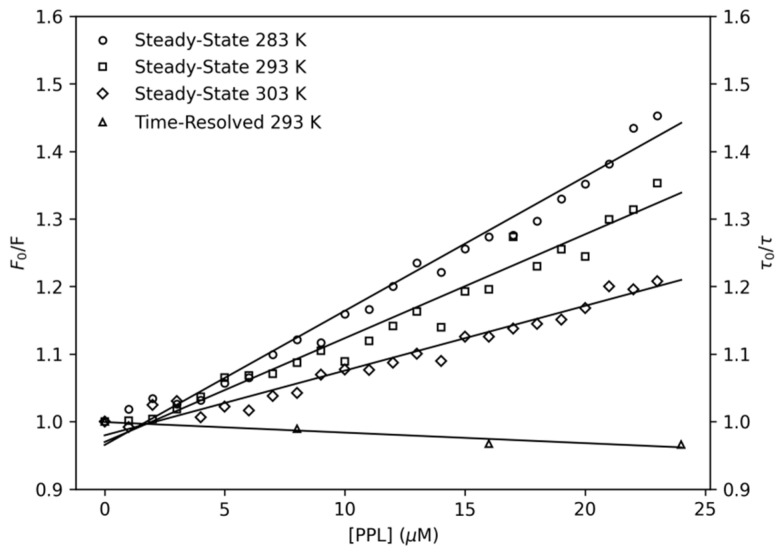
Left ordinate: Stern–Volmer plots at three temperatures, 283 K, 293 K, and 303 K. Right ordinate: Time-resolved fluorescence lifetime plot at 293 K; [*NBD*] = 4.0 μM, [*PPL*] = 0–24.0 μM. PPL was added to the sample in increments of 1.0 μM.

The Stern–Volmer plots (Figure 2) presented a linear response to the increment of PPL concentration. At temperatures 283 K, 293 K and 303 K, the *K_SV_* constant showed a noticeable decrease by the drop in slope of the linear regression, which is strong evidence of the static quenching process [24]. The quenching mechanism obtained by steady-state fluorescence results was confirmed by time-resolved experiments as a second experimental method. In this experiment, the NBD tryptophan lifetime of excited states were measured in the absence (*τ*_0_) and presence (*τ*) of different concentrations of PPL (Figure S1 and Table S1). The ratio (*τ*_0_/*τ*) of fluorescence lifetime, plotted at the right ordinate of Figure 2, remained close to unity and did not present equivalence with *F*_0_/*F*, which indicated that PPL poorly affects the NBD tryptophan fluorescence lifetime and confirms that the quenching mechanism is static [22]. To further confirm this result, the values of bimolecular constants *k_q_* were calculated, all *k_q_* are in the order of magnitude of 10^12^ M^−1^·s^−1^ which exceeded the limit of 10^10^ M^−1^·s^−1^ observed for dynamic quenching. In conclusion, these results characterize the quenching mechanism as static, which means that a complex has been formed by the PPL and NBD.

Table 1 shows the data for the Stern–Volmer constants and the bimolecular constant at different temperatures.

**Figure 3:**
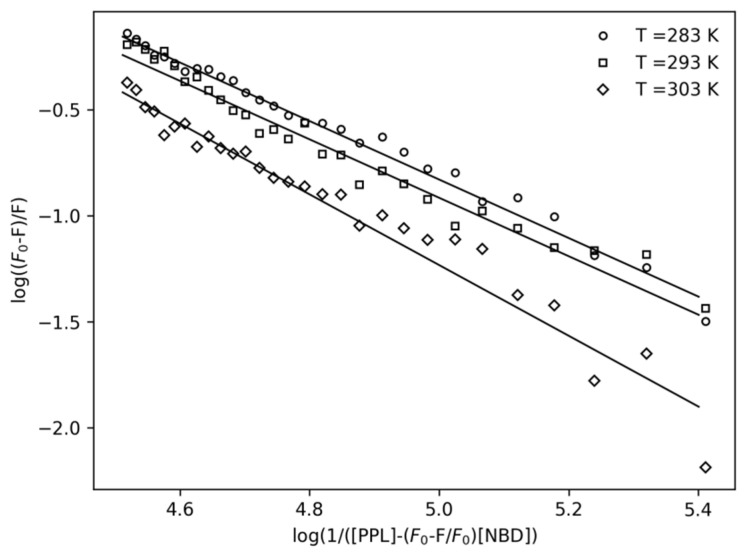
Double-log plots for the fluorescence quenching of NBD (4.0 μM) by the presence of PPL at 283 K, 293 K, and 303 K.

Once it was determined that a complex formation occurred, the association constant also known as binding constant (*K_a_*) was calculated. The variables *K_a_* were obtained by linearizing the function of the plot of Figure 3 using the double-logarithm equation (Equation (2)) that relates the quenching fluorescence intensities to the total concentration of PPL.

(2) $$log\;\left(\frac{F_{0}-F}{F}\right)=n\cdot log\;K_{a}-n\cdot log\;\left(\frac{1}{\left[PPL\right]-\left(\frac{F_{0}-F}{F_{0}}\right)\cdot \left[NBD\right]}\right)\;$$

The results of *K_a_* at different temperatures obtained for the first order model (*n*~1) are shown in Table 1. The binding constants found for different temperatures are in the order of magnitude of 10^4^ M^−1^. As shown in Table 1, the affinity of the complex is influenced by temperature, since the results of the binding equilibrium experiments showed that *K_a_* decreased while the temperature increased.

#### 2.1.1. Thermodynamic Parameters

**Figure 4:**
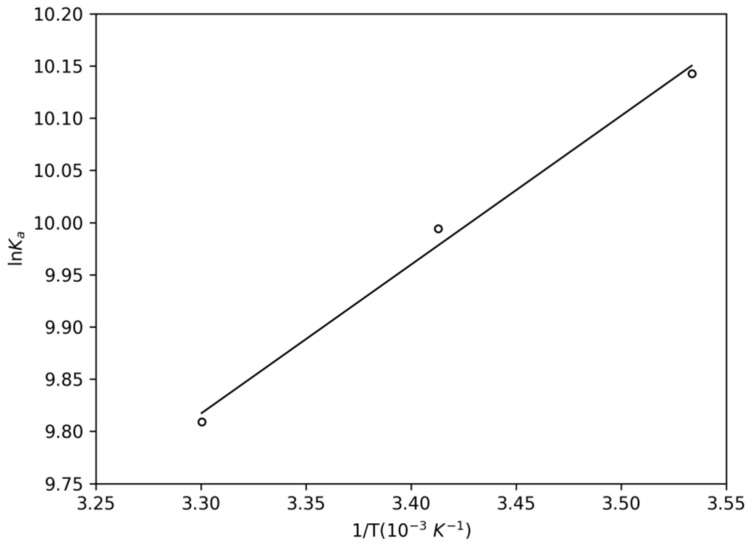
Van’t Hoff plot for the complex NBD-PPL at 283 K, 293 K, and 303 K.

To obtain a description of the thermodynamic of complex formation, the thermodynamic parameters ∆*S* (entropy variation) and ∆*H* (enthalpy variation) were determined by linear regression of the data shown in Figure 4, using the Van’t Hoff equation (Equation (3)). ∆*G* (Gibbs free variation) was obtained according to Equation (4).

(3) $$ln\;K_{a}=-\frac{\Delta H}{R\cdot T}+\frac{\Delta S}{R}$$

(4) ΔG=ΔH−TΔS

According to the results of the thermodynamic parameters gathered in Table 2, the values of ∆*G* exhibited negative values at the three temperatures, which showed the spontaneity of the complex formation process. Besides that, ∆*H* < 0 characterized the complexation as an exothermic process. Furthermore, the positive values of ∆*S* may be an effect of water molecules displacement due to PPL entrance into the protein [25]. The thermodynamic balance of ∆*H* and *T*·∆*S* indicated the Van der Waals interactions as the major contribution to the complexation [26].

#### 2.1.2. Interaction Density Function (IDF)

**Figure 5:**
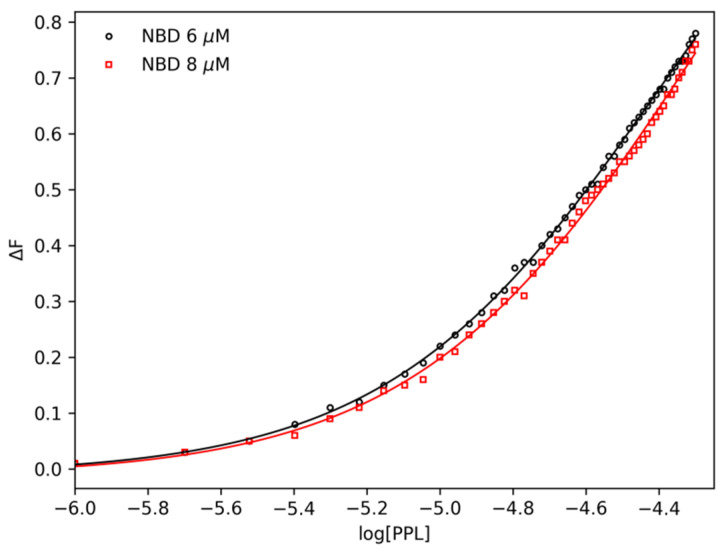
Plot of ∆*F* versus Log [*PPL*] obtained from PPL titration experiments with NBD concentrations of 6.0 μM and 8.0 μM at 293 K.

As a second method, IDF was also applied in fluorescence data in order to obtain a complete description of the system. Differently from the binding equilibrium model, IDF does not make use of any model a priori [27] and the advantage of applying IDF is the possibility of not only determining the number of binding sites but also identifying cooperativity occurrence among them. IDF considers that, if the free ligand concentration ([*PPL*]*_free_*) is the same for two at different concentrations of total protein ([NBD]), the average interaction density (Σ*υ_i_*) will also be the same, and consequently the system will have the same variation on the percentage of quenching (Δ*F*). The percentage of fluorescence quenching is given by Equation (5). Where *F* is the observed fluorescence signal in the presence of PPL and *F*_0_ is the observed fluorescence signal for free protein. Figure 5 shows the plot of Δ*F* per log [*PPL*] for two known concentrations of NBD adjusted by a sigmoidal function.

(5) $$\Delta F=\frac{\left|F-F_{0}\right|}{F_{0}}$$

Free ligand concentration and the average of interaction density are related to each other through the expression of mass conservation (Equation (6)).

(6) $$\left[PPL\right]={\left[PPL\right]}_{free}+\left(\sum \nu_{i}\right).\left[NBD\right]$$

**Figure 6:**
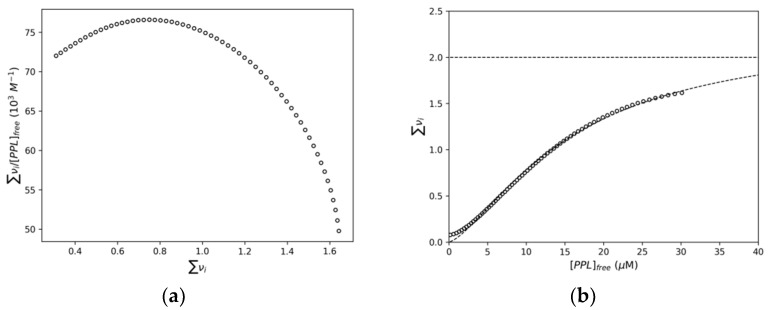
(**a**) Scatchard plot for the interaction of NBD and PPL obtained at 293 K based on IDF data. (**b**) Hill’s plot for the interaction of NBD and PPL.

By means of the plot shown on Figure 5, the values of [*NBD*] and [*PPL*] for each Δ*F* were obtained. According to the IDF results, a Scatchard plot was built (Figure 6a). This plot presented a concave function, revealing positive cooperativity between the NBD binding sites [28]. Interestingly, we reported recently that the interaction of piperine with NBD led to a cooperative mode of binding [29]. These results showed that different ligands can induce similar modes of binding in NBD structure.

The number of sites (*n*) and the binding constant (*K_b_*) were obtained using Hill’s model, based on Equation (7). Another parameter obtained through this model was the cooperativity, indicated by Hill’s coefficient (*h*). [30]

(7) $$\sum \nu_{i}=\frac{n\cdot {\left(K_{b}{\left[PPL\right]}_{free}\right)}^{h}}{1+\left(K_{b}{\left[PPL\right]}_{free}\right){\;}^{h}}$$

The Hill’s plot (Figure 6b) shows the variation of the average interaction density ($\sum \nu_{i}$) with the free ligand concentration (${\left[PPL\right]}_{free}$). With the mathematical fitting of these results, the number of sites (*n*) and the binding constant (*K_b_*) were found to be 2.2 ± 0.1 and (6.3 ± 0.2) × 10^4^ M^−1^, respectively. Besides that, the fitting also revealed the Hill’s coefficient (*h*) as 1.4 ± 0.1, confirming the cooperative binding of PPL toward NBD, previously detected in Scatchard plot.

These results showed that both methods applied in the analysis of fluorescence quenching (binding equilibrium model and IDF) are in agreement with respect to the order of magnitude of *K_b_*, since binding equilibrium model revealed the binding constant as (2.2 ± 0.5) × 10^4^ M^−1^.

### 2.2. Circular Dichroism

**Figure 7:**
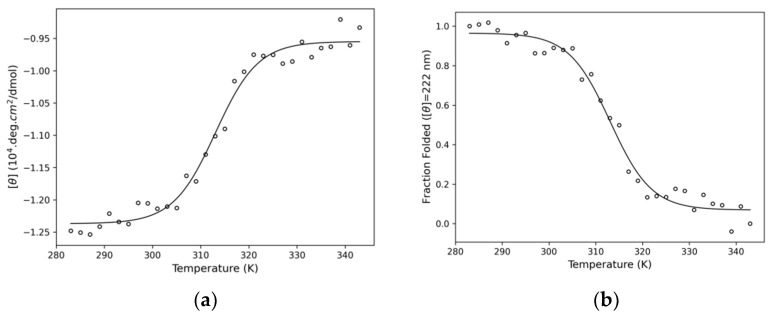
Denaturation curve of NBD monitored at 222 nm with a melting temperature of 314 K. (**a**) The plot shows the ellipticity [*θ*] versus temperature and (**b**) The plot shows the fraction folded calculated with Equation (12).

**Figure 8:**
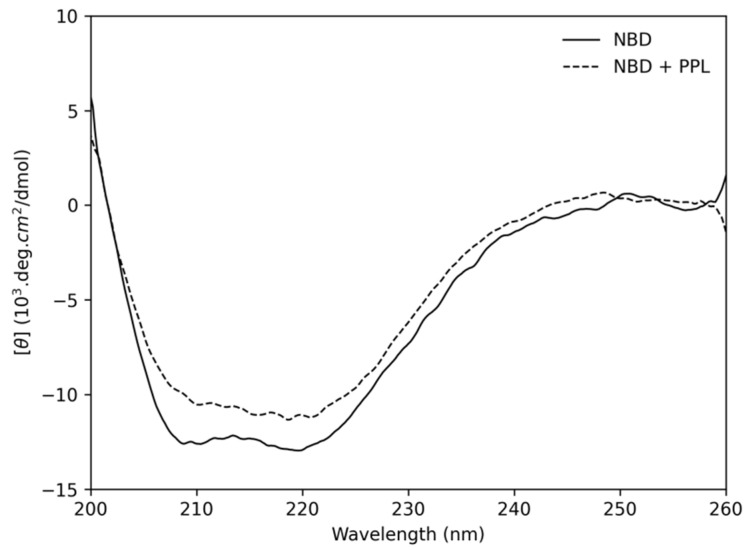
Circular Dichroism of free NBD (solid lines) and NBD + PPL at the stoichiometry 1:12 (dotted lines) at 293 K.

Circular Dichroism (CD) is a suitable method to analyse secondary structure of proteins in different conditions. In this way, CD experiments were performed to obtain both the thermal structural stability of the domain NBD (Figure 7) and possible secondary conformational changes due to the PPL interaction (Figure 8). Considering that NBD structure is predominantly composed by alpha-helices in solution with two CD characteristic bands centred at 208 nm (π-π*) and at 222 nm (n-π*) [31], the wavelength of 222 nm was followed to monitor the thermal transition (Figure 7b) of NBD. According to the results obtained (Figure 7b), NBD experienced a transition from folded to unfolded state at ~314 K. Similar results were obtained from calorimetric and spectroscopic methods applied to bacterial HSP (DnaK) [32,33].

Figure 8 shows the CD spectrum of pure NBD and in the presence of PPL at stoichiometry of 1:12, the same stoichiometry reached in the IDF experiment. According to the results, the deconvolution of pure NBD spectrum presented 38% of alpha-helices, 14% of β-sheet, 20% of turn and 28% of coil, which is in a good agreement with the results obtained by Zazeri et al. [29] at a similar temperature. After PPL addition, NBD underwent some secondary conformational changes, being 33% of α-helices, 17% of β-sheet, 19% of turn and 31% of coil. Interestingly, while the percentage of α-helices decreased by 5%, the percentage of β-sheet increased by 3%. Despite the smaller secondary structural changes found in this work compared to those reported by Zazeri et al. [29] for the interaction of NBD and piperine, the same behavior for α-helices and β-sheet changes were observed. Furthermore, although the results showed a maximum secondary structure change of 4% (related to α-helices), it is not statistically significant in terms of conformational changes, such changes may modulate the biochemical activity [34].

### 2.3. Molecular Docking

**Figure 9:**
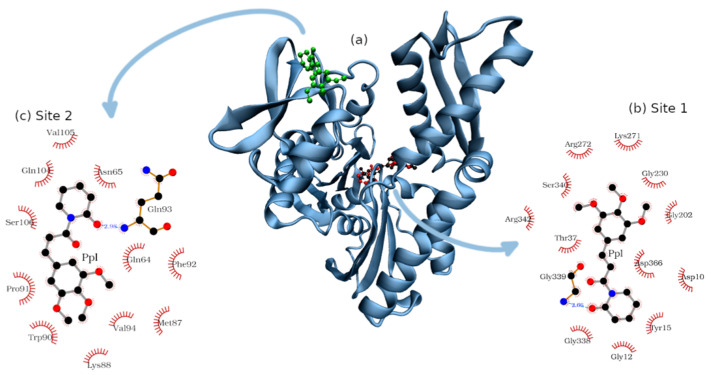
Bind sites of NBD accessed by PPL: (**a**) NBD structure with PPL in Site 1 (red) and in Site 2 (green), in this picture ADP molecule is represented in black. (**b**) The amino acids composition of Site 1 and the interactions between PPL and NBD, where one hydrogen bond was found between Gly339 and PPL (blue dashed line). (**c**) The amino acids composition of Site 2 and the interactions between PPL and NBD, where one hydrogen bond was found between Gln93 and PPL (blue dashed line).

The structure of NBD obtained from PDB 1S3X was directly subjected to molecular docking that disclosed several possible pockets where PPL can interact with NBD (Figure S2), with energy ranked from (7.2 to 5.0 kcal). The analysis of the poses from cluster “a” to “g” (Figure S3) revealed that they are in the environment of the binding site accessed by the ADP molecule, represented in black. The next cluster analyzed was “h”, which is not in the proximity of the ADP binding site, as shown in Figure S3. The next clusters were not considered once they were less populated than the previous ones. For the next analyses, we will consider the coordinates from cluster “a” and “h” as being Site 1 and 2, respectively (Figure 9).

The composition of the bind sites was organized in Table 3, which shows that Site 1 is rich in glycine and presents a generous number of polar amino acids (whether charged or not). Ligplot software detected a hydrogen bond between PPL and the nitrogen atom of Gly339 which is part of the backbone of NBD. It is worth noting that such analysis was made based on a fixed pose calculated by molecular docking; a more holistic analysis of hydrogen bonds will be presented based on molecular dynamics results.

Site 2 presents an equilibrated balance of polar and non-polar amino acids. No negatively charged residues were found in the environment, which favors the interaction with PPL with protein since its structure presents charge delocalization that concentrates negative charges in the extremities of the molecule [35]. Besides that, the interaction of PPL with Site 2 includes one hydrogen bond with Gln93.

### 2.4. Molecular Dynamics

**Figure 10:**
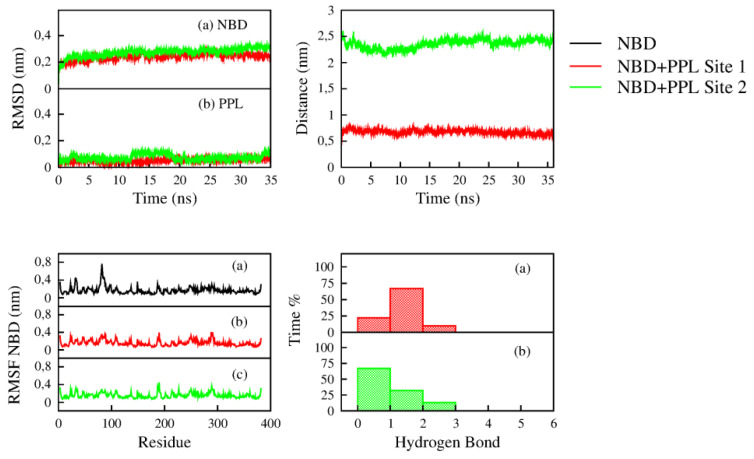
Parameters obtained from the molecular dynamics simulation of the NBD (black), NBD with PPL in Site 1 (red) and Site 2 (green). **Top Left**: The RMSD of (**a**) protein NBD and (**b**) molecule PPL. **Top Right**: Distance between COG of NBD and COG of PPL in Sites 1 and 2. **Bottom Left**: The RMSF of the residues of (**a**) NBD, (**b**) NBD when interacting with PPL in Site 1 and (**c**) NBD when interacting with PPL in Site 2. **Bottom Right**: The hydrogen bonds performed between NBD and PPL in (**a**) Site 1 and (**b**) Site 2.

The equilibration and stability of the complexes formed by NBD and PPL in Sites 1 and 2 was verified through the parameters obtained from molecular dynamics (Figure 10). The root mean square deviation (RMSD) of NBD with PPL in Site 1 and 2 remained stable during the simulation, fluctuating around 0.25 nm. Similar behavior was found to the RMSD calculated for PPL atoms when bound in Site 1 and 2, which remained stable, fluctuating around 0.1 nm, i.e., PPL remained in the Sites 1 and 2 throughout the simulation.

Another helpful parameter used to verify the stability of the protein-ligand complex is the distance from the centre of geometry (COG) of protein to the COG of ligand. The distance calculated for the complex formed by NBD and PPL in Site 1 remained around 0.7 nm, as expected, since the molecule is located close to the centre of geometry of NBD (Figure 9). For Site 2, the distance fluctuated around 2.5 nm, confirming that PPL remained in the binding site located in the periphery of NBD (Figure 9).

The root mean square fluctuation (RMSF) of the residues of NBD free, bound to PPL in site 1 and 2 was calculated to verify possible changes in the dynamics of the protein caused by the interaction with ligand. No drastic change was observed in the RMSF of NBD with PPL in Sites 1 and 2 when compared to RMSF of free NBD. A slight change was observed for residues 78–87 that compose an α-helix close to Site 1. However, the change in the dynamics is not necessarily due to the interaction of PPL in Site 1 since the dynamics of such residues also presented low fluctuation in the analysis of NBD with PPL in Site 2.

The analyses of hydrogen bonds showed that in Site 1 PPL performs between 1 and 2 H-bond with residues of NBD 72% of the simulation time. Hydrogen bonds are less observed in the interaction of PPL with Site 2. Molecular dynamics revealed that the number of the H-bond was between 0 and 1 in 73% of the simulation time. Hydrogen bonds give an enthalpic contribution to the thermodynamic balance. Application of the Van’t Hoff model to the experimental data (Table 2) gave insight into an equilibrated balance between entropic and enthalpic interactions. Molecular dynamics corroborated this result since a moderate formation of H-bonds in both sites was detected.

**Figure 11:**
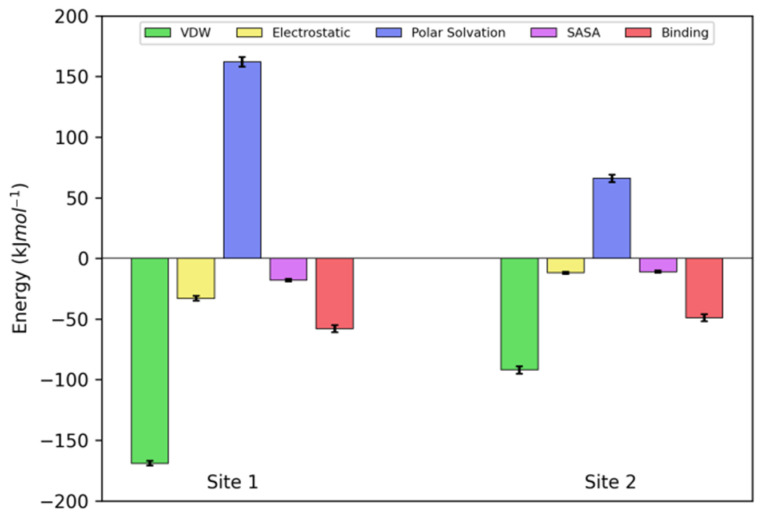
Energy decomposition obtained by MMPBSA for the interactions of NBD with PPL for Site 1 and 2. Contributions of Van der Waals (VDW), electrostatic, polar solvation (P.Solvation), and solvent accessible area (SASA) to the final binding energy (Binding).

Figure 11 shows the results of MMPBSA calculations applied to the trajectory from molecular dynamics simulations. According to the results, the binding sites have similar binding energy (−58 ± 3 kJ·mol^−1^ and −49 ± 3 kJ·mol^−1^ for Site 1 and 2, respectively). Moreover, the results revealed the Van der Waals as the predominant interaction of the complex, which reinforced the result obtained from Van’t Hoff analyses.

## 3. Materials and Methods

### 3.1. Reagents

Piperlongumine (>97%) was purchased from Sigma-Aldrich Chemical Co. (Schnelldorf, Germany), as dibasic sodium phosphate (>99%) reagents, anhydrous citric acid (>99%), and sodium chloride (>99%). Lyophilised Nucleotide Binding Domain of Heat Shock Protein 70 kDa (>97%) was purchased from GenScript. Methanol was purchased from Dynamics Química Contemporânea LTDA (Indaiatuba, SP, Brazil). All the materials purchased were used as supplied. Ultrapure water was prepared by a Millipore water purification system -Direct-Q UV-3(Merck KGaA, Darmstadt, Germany). Lyophilized NBD was reconstituted in a 50 mM phosphate buffer containing 150 mM sodium chloride, and the pH was adjusted to 7.4 with anhydrous citric acid. Stock solutions of PPL were prepared in methanol. The concentrations of PPL and NBD solutions were determined by UV-Vis experiments performed on Biospectro spectrophotometer (Biospectro, Curitiba, PR, Brazil), using the extinction coefficient of 18700 M^−1^·cm^−1^ at 326 nm for PPL and 20,525 M^−1^·cm^−1^ at 280 nm for NBD.

### 3.2. Experimental Methods

#### 3.2.1. Steady-State Fluorescence Spectroscopy

Fluorescence experiments were performed on the Lumina (Thermo Fisher Scientific, Waltham, MA, USA) stationary state spectrofluorimeter equipped with thermal bath and Xenon lamp. A 100 μL quartz cuvette with 2 mm × 10 mm optical path was used in the experiments. The widths of the excitation and the emission slits were adjusted to 10 nm. The wavelength of 295 nm was used to excite the single tryptophan residue of NBD (Trp90). The emission spectra were obtained in the range from 305 to 500 nm with a resolution of 1.0 nm ± 5.0 nm. Each emission point collected was the average of 15 accumulations. The software ScanWave was used to collect the measured data.

In the binding equilibrium experiments, aliquots of PPL (increment of 1.0 μM) were added in NBD solution at 4.0 μM. Measurements were performed at 283 K, 293 K, and 303 K. In the interaction density function analysis, aliquots of PPL (increments of 1.0 μM) were added in NBD solutions at 6.0 μM and 8.0 μM at a fixed temperature (293 K). In all experiments, the final volume of methanol in the buffer was <1.0%.

The correction of the inner filter effects was done with Equation (8), where *F_corr_* and *F_obs_* are corrected and observed fluorescence intensities, and *A_ex_* and *A_em_* are the absorbance of the sample in a 10 mm optical path cuvettes at the excitation and the emission wavelengths, respectively [22].

(8) $$\;F_{corr\;}=F_{obs}\;\cdot 10^{\frac{\left(5\cdot A_{ex}+A_{em}\right)}{10}}$$

#### 3.2.2. Time-Resolved Fluorescence

Fluorescence lifetime measurements were performed using a Mini-tau filter-based fluorescence lifetime spectrometer coupled to a Time-Correlated Single Photon Counting (TCSPC) system (Edinburgh Instruments, Livingston, UK). Aliquots of PPL were added in the NBD solution from 0 to 24 μM. Experiments were carried out at 293 K.

The sample was excited at 295 nm using a picosecond pulsed light emitting diode (LED), and fluorescence decay was collected using a 340 nm filter. The fluorescence decay profile (Figure S1) was fitted using multiexponential decay (Equation (9)), where *τ_i_* is the lifetime of each component, and *α_i_* is the contribution of each component to total fluorescence decay. The average lifetime <*τ_avg_*> was calculated using Equation (10) (Table S1).

(9) $$I_{T}=\sum_{i=1}^{n}\alpha_{i}.e^{\frac{-T}{\tau_{i}}}$$

(10) $$\;\tau_{avg}=\frac{\alpha_{1}\tau_{1}{}^{2}+\alpha_{2}\tau_{2}{}^{2}}{\alpha_{1}\tau_{1}+\alpha_{2}\tau_{2}}$$

#### 3.2.3. Circular Dichroism

Circular dichroism spectra were recorded on a Jasco J-815 spectropolarimeter model DRC-H (Jasco, Easton, MD, USA) equipped with a demountable quartz cell with a 0.01 cm optical path length. The CD spectra were recorded from the 200 to 260 nm range with a scan rate of 20 nm/min and a spectral resolution of 0.1 nm. For each spectrum, 15 accumulations were performed. For the denaturation experiments, the spectra were recorded in the temperature range of 280 and 340 K. The ellipticity *θ* collected in millidegrees was converted to mean residue ellipticity [*θ*] (deg·cm^2^·dmol^−1^) using Equation (11) and the protein denatured fractions were determined with Equation (12) where [*θ*]*^nat^* is the [*θ*] at 280 K and [*θ*]*^den^* is [*θ*] at the 340 K. For the interaction experiments, the molar ratio NBD:PPL was 1:12, the buffer spectrum was subtracted, and the temperature was kept at 293 K. The secondary structures percentages were calculated with CDPro applying the CONTIN method with the SP43 protein library [36].

(11) $$\left[\theta \right]=\frac{\theta \left(mdeg\right)}{10\cdot \left[P\right]\cdot l\cdot n}$$

(12) $$f=\frac{\left({\left[\theta \right]}^{obs}-{\left[\theta \right]}^{den}\right)}{\left({\left[\theta \right]}^{nat}-{\left[\theta \right]}^{den}\right)}$$

### 3.3. Computational Methods

#### 3.3.1. Molecular Docking

PPL structure used in molecular docking was obtained from ab initio calculations from our previous work [31]. The AutoDockTools [37] software of the MGL program Tools 1.5.4 was used to prepare the NBD (PDB 1S3X) by adding polar hydrogen atoms and Gasteiger charges. The maps were generated by the AutoGrid 4.2 program [38] with a spacing of 0.541 Å, a dimension of 126 × 126 × 126 points, and grid center coordinates of 51.315, 42.946, and 49.437 for x, y, and z coordinates, respectively. The AutoDock 4.2 program [37] was used to investigate the NBD binding sites using the Lamarckian Genetic Algorithm (LGA) with a population size of 150, a maximum number of generations of 27,000, and energy evaluations equal to 2.5 × 10^6^. All other parameters were selected as software defaults. To generate different conformations, the total number of runs was set to 100. (Figure S2). The final conformations were visualized on VMD [39]. The binding microenvironment was generated by LigPlot [40].

#### 3.3.2. Molecular Dynamics

The simulations of the complex NBD/PPL were performed with GROMOS53a6 force field [41] by Gromacs v.5.1.4 [42]. The complex was placed in a rectangular box, solvated with the simple point charge water (SPC) [43] and neutralized with NaCl in a concentration of 150 mM. The energy minimization was performed with the steepest descent algorithm with 5000 steps and a tolerance of 10 kJ·mol^−1^. The cut-off for small-range interactions was set to 10 Å and the long-range electrostatic interactions were treated with particle mesh Ewald (PME) [44]. Then, the heavy atoms were restrained with a force constant of 1000 kJ mol^−1^ nm^−2^ and the system was submitted to the first stage of equilibration for 100 ps in the NVT ensemble coupled to V-rescale thermostat at 293 K [45]. All bonds were constrained with the LINCS algorithm [46]. Random velocities were generated by the Maxwell–Boltzman distribution. The second stage of equilibration was performed in the NPT ensemble for 100 ps of simulation coupled to Parrinello–Rahman barostat [47] at 1 atm. Finally, the restrictions were turned off and the molecular dynamics simulations were performed with steps of 2 fs using the leap-frog algorithm to integrate the equations of motion. The hydrogen bonds were calculated by *gmx hbond*. The results presented are an average of three independent simulations.

The free energy of the binding process of PPL toward NBD was calculated by G_mmpbsa tool [48], using the molecular mechanics Poisson–Boltzmann surface area (MM/PBSA) method applied to the snapshots obtained from molecular dynamics simulations. The snapshots were extracted in intervals of 500 ps from the trajectory after the system reached the equilibrium, which was verified by the root mean square deviation (RMSD) and the distance from the center of geometry of NBD to PPL, obtained by the programs *gmx rms* and *gmx distance*, respectively (Figure 10). The coarse grid-box (cfac) was set as 2 and the finer grid-box (fadd) was set as 20. The concentration of positive and negative ions was set as 0.150, being the positive and negative radii set as 0.95 and 1.81 Å, which correspond to sodium and chloride atoms, respectively. The values for the vacuum (vdie) and solvent (sdie) dielectric constants were set and 1 and 80, respectively. The solute dielectric constant (pdie) was set as 4.

## 4. Conclusions

Multi-pronged spectroscopic analyses aided by computational modeling elucidated in detail the main features of the NBD/PPL interaction for the first time. Steady state fluorescence spectroscopy and time-resolved fluorescence results revealed the complex formation via static quenching mechanism. The use of binding equilibrium and IDF methods to treat the fluorescence quenching resulted in a binding affinity with an order of magnitude of 10^4^ M^−1^. Besides that, IDF method revealed two cooperative binding sites for PPL in NBD. Van’t Hoff analyses showed through the thermodynamic balance that the complexation between NBD and PPL is an exothermic and spontaneous process, with Van der Waals as the key interaction to stabilize the complex. Molecular docking and molecular dynamics disclosed the main features of the microenvironments of interaction. In this context, the microenvironments are rich in polar (charged or non-charged) amino acids. Moreover, MMPBSA data reinforced the experimental results, confirming the equivalence of the binding sites and that Van der Waals interactions were predominant in the complex interaction. Although the environments disclosed by the analyses are highly polar, an elevated number of H-bonds was not observed. Further chemical modifications on the PPL structure aimed at increasing the affinity for the NBD binding sites would benefit from the insertion of hydrogen bond donors to reach the acceptors present in these sites. In conclusion, this work brings the key aspects involved in NBD and PPL interaction, which will further the drug development of PPL.

## Figures and Tables

**Table 1:** Stern–Volmer constant (*K_SV_*), bimolecular constants (*k_q_*) and binding constant (*K_a_*) for the complex NBD and PPL at 283, 293 and 303 K.

*T* (K)	*K_SV_* (×10^4^ M^−1^)	*K_q_* (×10^12^ M^−1^·s^−1^)	*K_a_* (×10^4^ M^−1^)
283	1.9 ± 0.1	8.9 ± 0.1	2.5 ± 0.8
293	1.5 ± 0.1	6.9 ± 0.1	2.2 ± 0.5
303	1.0 ± 0.1	4.3 ± 0.1	1.8 ± 0.2

**Table 2:** Thermodynamic parameters of the complex NBD-PPL at the temperatures of 283, 293 and 303 K.

*T* (K)	∆*G* (kJ·mol^−1^)	∆*H* (kJ·mol^−1^)	∆*S* (J·mol^−1^·K^-1^)	*T*·∆*S* (kJ·mol^−1^)
283	−23.7 ± 1.3	−11.9 ± 0.9	42.4 ± 3.4	11.9 ± 0.9
293	−24.2 ± 1.3			12.4 ± 0.9
303	−24.6 ± 1.3			12.8 ± 1.0

**Table 3:** The characteristics of amino acids that compose the microenvironment of Sites 1 and 2 obtained by molecular docking.

Binding Sites	Amino Acids			
	Non-Polar	Positively Charged	Negatively Charged	Polar
1	Gly12, 202, 230, 338, 339	Lys271; Arg272, 342	Asp10, 366	Tyr15; Thr37; Ser340
2	Val94, 105; Met87; Pro91; Trp90; Phe92	Lys88	-	Asn65; Gln64; 93, 104; Ser106

## Data Availability

Data is contained within the article and Supplementary Materials.

## Supplementary Materials

The following are available online at https://www.mdpi.com/article/10.3390/ph14121298/s1, Figure S1: Time-dependent fluorescence decay of NBD with PPL concentration range from 0 to 24 µM. [NBD] = 10 μM, T = 298 K and λexc = 295 nm, Figure S2: Molecular docking clusters with their respective binding energy scores, Figure S3: Representation of the clusters a-h calculated by molecular docking. ADP molecule is represented in black, cluster a, b, c, d, e, f, g and h are represented in red, yellow, silver, tan, blue, orange and green, respectively, Table S1: Tryptophan lifetime in different stoichiometries HSP70:PPL obtained through biexponential decay.
