# Supplementary material for: Unravelling the Interaction of Piperlongumine with the Nucleotide-Binding Domain of HSP70: A Spectroscopic and In Silico Study
Source: Pharmaceuticals (Basel). 2021 Dec 13;14(12):1298. doi: 10.3390/ph14121298 (PMC8703466; doi:10.3390/ph14121298)
Supplement: Supplementary file 1 [file pharmaceuticals-14-01298-s001.zip › pharmaceuticals-1473599-supplementary.pdf]

pharmaceuticals

MDPI

Supplementary material

Unravelling the Interaction of Piperlongumine with the Nucleotide-Binding Domain of HSP70: A Spectroscopic and In Silico Study

Ana Paula Ribeiro Povinelli[1], Gabriel Zazeri[1], Alan M. Jones[2,*] and Marinônio Lopes Cornélio[1,*]

[1] Departamento de Física, Instituto de Biociências, Letras e Ciências Exatas (IBILCE), UNESP, Rua Cristovão Colombo 2265, CEP 15054-000, São José do Rio Preto, SP, Brazil; gabriel.zazeri@unesp.br (G.Z.); ana.povinelli@unesp.br (A.P.R.P.)
[2] School of Pharmacy, University of Birmingham, Edgbaston B15 2TT, UK
* Correspondence: a.m.jones.2@bham.ac.uk (A.M.J.) and m.cornelio@unesp.br (M.L.C.)

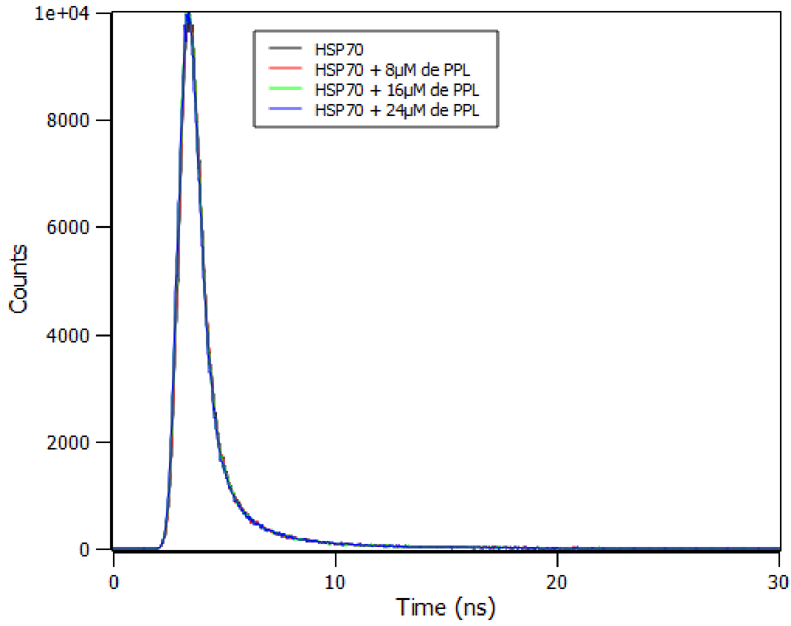

Figure S1. Time-dependent fluorescence decay of NBD with PPL concentration range from 0 to 24 μM . [NBD] = 10 μM, T = 298 K and λ_{exc} = 295 nm.

Table S1. Tryptophan lifetime in different stoichiometries HSP70:PPL obtained through biexponential decay.

[PPL] (μM)	α1	τ1 (ns)	α2	τ2 (ns)	τavg (ns)
0	0.78	0.72	0.23	3.34	2.22
8	0.77	0.71	0.23	3.34	2.24
16	0.79	0.75	0.20	3.58	2.29
24	0.77	0.71	0.23	3.40	2.30

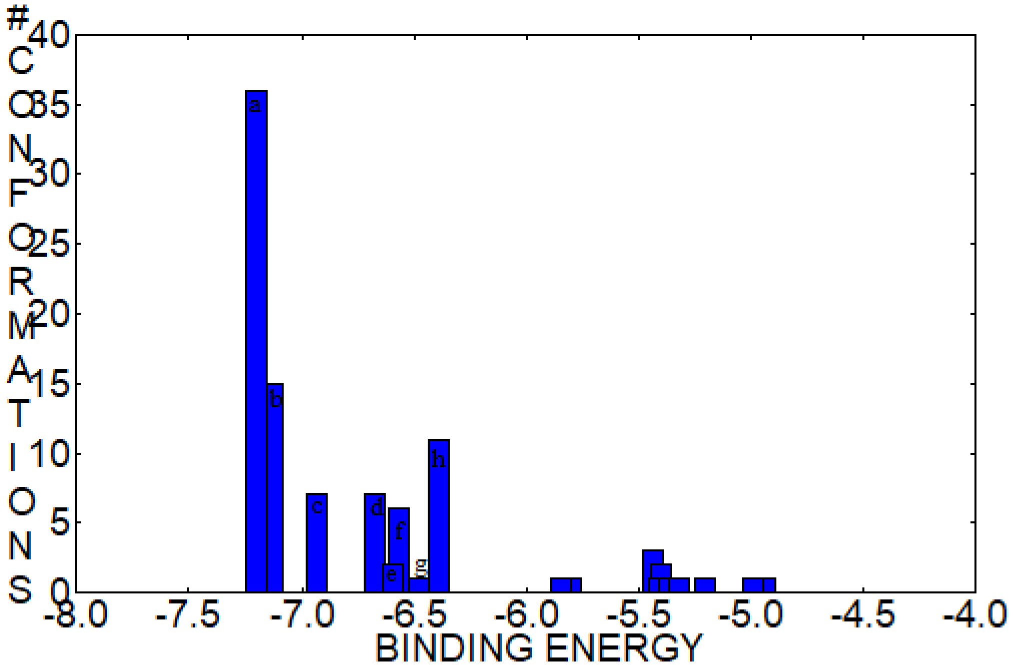

Figure S2. Molecular docking clusters with their respective binding energy scores.

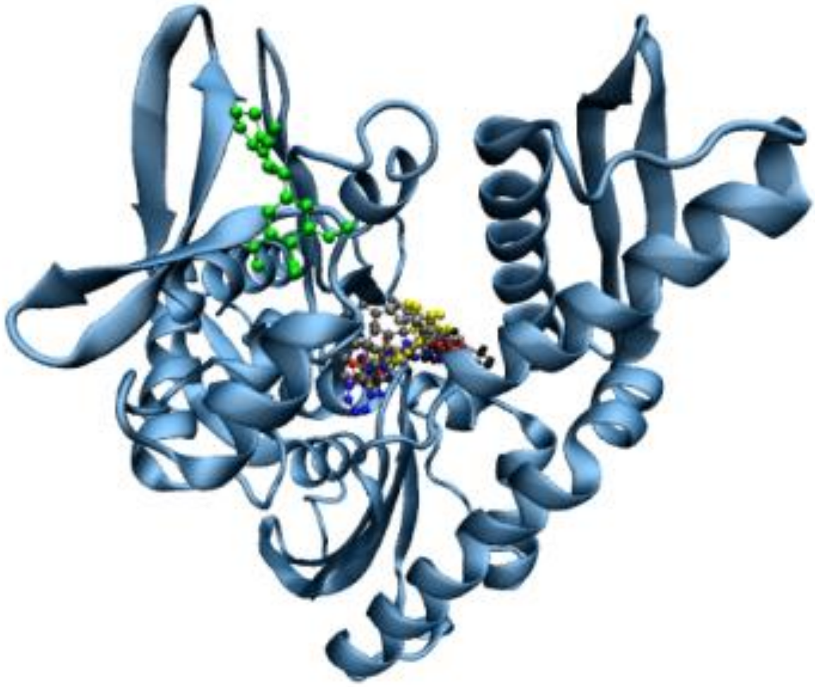

Figure S3. Representation of the clusters a-h calculated by molecular docking. The molecule ADP is represented in black, cluster a, b, c, d, e, f, g and h are represented in red, yellow, silver, tan, blue, orange and green, respectively.
